# Krisenthemen in Familien zu Beginn der COVID-19-Pandemie: „Homeoffice mit Kindern ist wie Zähneputzen mit Nutella“

**DOI:** 10.1007/s12592-022-00414-8

**Published:** 2022-05-17

**Authors:** Sabine Andresen, Johanna Wilmes

**Affiliations:** Johann Wolfgang Goethe-Universität, Frankfurt am Main, Frankfurt am Main, Deutschland

**Keywords:** Eltern, Mütter, Belastung, Krise, COVID-19, Parents, Mothers, Stress, Crisis, COVID-19

## Abstract

Während der COVID-19-Pandemie in Deutschland standen besonders Familien mit Kindern vor großen Herausforderungen. Der erste Lockdown erscheint im Zeitverlauf markant, da sich das ausdifferenzierte Familienleben fast ausschließlich auf die häusliche Umgebung konzentrierte und Bildungs- und Betreuungsstätten geschlossen waren. Das Wegbrechen der öffentlichen Infrastruktur definieren wir hier als zentrales Krisenphänomen für Familien.

Im Fokus dieses Beitrags steht eine Auswertung von 5075 Kommentaren aus dem Online-Fragebogen der Studie „KiCo – Kinder, Eltern und ihre Erfahrungen während der Corona-Pandemie“, an der im April/Mai 2020 über 25.000 Eltern mit Kindern unter 15 Jahren teilnahmen. Hauptsächlich stammen die Kommentare von Müttern zwischen 30 und 50 Jahren, die zum Zeitpunkt der Befragung größtenteils im Homeoffice arbeiteten und ein bis zwei Kinder unter 15 Jahren hatten.

Anhand der Kommentare können wir nachzeichnen, wie das Krisenerleben verhandelt wurde bzw. wie auf das Narrativ der Krise in den ersten zwei Monaten der Pandemie in Deutschland Bezug genommen wurde. Folgende Kategorien konnten identifiziert werden: Die Dauer der Krise (Zeit), Krise als Chance für gesellschaftlichen Wandel, die Krise der Demokratie, Krise als Chance für die Familie, Leidtragende der Krise.

## Abstract (en)

During the coronavirus disease 2019 (COVID-19) pandemic in Germany, families with children, in particular, were faced with a huge challenge. Over the course of time the first lockdown seemed striking, as the differentiated family life was practically exclusively concentrated on the domestic environment and educational and care facilities were closed. The curtailing of the public infrastructure is defined here as the core crisis phenomenon for families. The focus of this article is on the evaluation of 5075 comments from the online questionnaire on the study “KiCo—Children, parents and their experiences during the corona pandemic”, in which over 25,000 parents with children under 15 years old participated in April and May 2020. The comments are mostly from mothers aged 30–50 years old, the majority of whom were working from home at the time of the survey and had 1–2 children.

Based on the comments, it could be reconstructed how the crisis experience was dealt with and how the narrative of the crisis was referred to in the first 2 months of the pandemic in Germany. The following categories could be identified: the duration of the crisis (time), crisis as a chance for change in society, the crisis of democracy, crisis as a chance for the family and the sufferers of the crisis.

## Einleitung

Der globale Ausbruch der Pandemie erschien in Deutschland zunächst als fernes Ereignis. Doch mit den ersten staatlichen Maßnahmen zur Eindämmung des Infektionsgeschehens war der Alltag nahezu jeder Familie betroffen. Gleichwohl gab es neben Gemeinsamkeiten auch deutliche Unterschiede im Erleben des ersten Lockdowns: So haben nicht alle die unfreiwillige „Auszeit“ als existenzielle Krise, sondern als befristete Befreiung von Freizeitstress, Work-Life-Balance-Problemen und Ansprüchen an perfektes Auftreten erlebt (Andresen et al. 2020b). Dort, wo Ressourcen wie Geld, aber auch Zeit für Erholung und Wohnraum bereits vor Ausbruch von COVID-19 knapp bemessen waren, hat die Summe der Infektionsschutzmaßnahmen allerdings zu einem krisenhaften Alltag geführt (Eckardt 2021). Insbesondere aber standen Familien mit Kindern und Jugendlichen vor der Herausforderung, das ausdifferenzierte moderne Leben – Arbeit, Betreuung, Förderung von Familienmitgliedern mit besonderen Bedarfen, Unterricht und Bildung, Freizeit und Verpflegung, Fitness und Beziehungspflege – nur noch innerhalb der häuslichen Umgebung zu gestalten und zu organisieren. Für Familien und Familienmitglieder brachen mit dem ersten Lockdown wesentliche Bestandteile der öffentlichen Infrastruktur weg, davon zeugt das Nutella-Zitat der Überschrift. Diesen Sachverhalt definieren wir im Folgenden als zentrales Krisenphänomen für Familien. Die ergriffenen Infektionsschutzmaßnahmen hatten eine Verdichtung in der häuslichen Umgebung und die Konzentration vieler sozialer Praktiken auf den familiären Wohnraum zur Folge. Dadurch standen Mütter und Väter, insbesondere bei gleichzeitiger Erwerbstätigkeit, vor erhöhten Anforderungen im Alltag.

Ausgehend von dieser hier vorgenommenen Krisendiagnose stellt sich die Frage, wie Mütter, Väter, Großeltern, Kinder und Jugendliche den Anfang der Pandemie erlebten und was von ihnen als krisenhaft empfunden wurde.

Auf diese und angrenzende Fragen hat nicht nur in Deutschland, sondern in vielen anderen Ländern die sozialwissenschaftliche Forschung erste Antworten zu geben versucht. Teils empirisch fundierte Einschätzungen zu sozialen, ökonomischen, emotionalen und psychischen Folgen der Maßnahmen zur Eindämmung des Infektionsgeschehens auf Kinder, Jugendliche, Mütter und Väter lagen schon 2020 vor. Der mediale öffentliche Diskurs in Deutschland hat zudem während des ersten Lockdowns den Umgang mit Kindern und Jugendlichen und Auswirkungen auf deren Entwicklung, Bildung und psychosoziale Gesundheit problematisiert.

Dieser Beitrag setzt bei den ersten Deutungen von Eltern mit Kindern unter 15 Jahren an und wirft den Blick zurück auf den Anfang der COVID-19-Pandemie als eine Art „Einschlagereignis“ (Assmann 2014) für die Gesamtbevölkerung, aber insbesondere für Familien und deren Routinen und Beziehungen zwischen den Generationen im Frühjahr 2020. Ausgehend von einer Onlinebefragung, „Kinder und Jugendliche in Corona“ (KiCo), die im Mai 2020 bundesweit durchgeführt wurde, geht es um den Blick von Müttern und Vätern auf sich selbst, ihre Kinder, ihre Familie, auf Erwerbsarbeit, das Betreuungs- und Bildungssystem sowie auf die Gesellschaft in dieser Anfangsphase.

Im nächsten Abschnitt werden im Sinne einer kurzen Erinnerung die Maßnahmen im Zuge des ersten Lockdowns in Deutschland thematisiert. Ohne Anspruch auf Vollständigkeit erfolgt diese mit Blick auf die Infrastruktur für Familien. Im dritten Abschnitt geht es um ausgewählte Themenfelder des internationalen Forschungsstandes, aus denen sich relevante Aspekte zu Krisen in Familien und für Familienmitglieder ableiten lassen. Auch hier beansprucht die Darstellung keine Vollständigkeit, sondern zielt auf einen Sensibilisierungsrahmen für die empirische Analyse. Der vierte Abschnitt enthält die für diesen Argumentationsgang relevanten methodischen Informationen zu der gemeinsam mit Kolleg:innen der Universität Hildesheim durchgeführten KiCo-Studie sowie zum erzielten Sample. Im fünften Abschnitt werden die Ergebnisse einer Teilauswertung der frei formulierten Kommentare von Müttern und Vätern beschrieben und im sechsten Abschnitt abschließend diskutiert.

## Zum „Anfang“ des COVID-19-bezogenen Infektionsschutzes in Deutschland

Von einem so gefährlichen Infektionsgeschehen auch in Europa betroffen zu sein, hat in dieser Zeit die Mehrheit der europäischen Länder und ihrer Bürger:innen 2020 vergleichsweise überraschend und unvorbereitet getroffen. Die Kontaktbeschränkungen und die Schließung der für Familien relevanten Infrastruktur in den Bereichen Betreuung, Bildung, Freizeit, Beratung, spezifische Förderung und das Verbot, öffentliche Spielplätze zu nutzen und sich mit mehreren Personen aus verschiedenen Haushalten privat zu treffen oder sich an öffentlichen Plätzen aufzuhalten, haben sofort Konsequenzen für den Alltag in Familien nach sich gezogen. Die restriktiven Kontaktbeschränkungen zu den älteren Angehörigen in Pflegeeinrichtungen sowie die Empfehlung, Großeltern aufgrund der Infektionsgefahr nicht in die Kinderbetreuung einzubeziehen, führte zu weiteren Herausforderungen – etwa die Kommunikation zur Großmutter im Pflegeheim aufrecht zu erhalten oder die Kinderbetreuung zu organisieren, weil viele Großeltern dabei zentrale Aufgaben übernehmen^1^.

Für viele Mütter und Väter hat sich zudem das Erwerbsarbeitsleben verändert: Wer konnte, sollte im Homeoffice tätig sein, unabhängig von technischer Ausstattung und Platz zu Hause. Nicht in allen Berufszweigen war und ist Homeoffice möglich, sodass in vielen Produktionsbetrieben Arbeitnehmer:innen weiterhin zur Arbeit gependelt sind, obwohl es keine oder nur eine unzureichende Betreuung zu Hause gab. Körpernahe Dienstleistungen wurden eingestellt, die Gastronomie geschlossen und viele aus diesen und anderen Branchen wurden in Kurzarbeit geschickt^2^. Ein Teil der Mütter und Väter in Deutschland musste sich im Frühjahr 2020 auch arbeitslos melden^3^. Dem gegenüber kamen auf diejenigen in sogenannten „systemrelevanten“ Berufsgruppen erhebliche Mehrbelastungen hinzu.

Im öffentlichen Diskurs dieser Zeit war die Situation von Müttern und Vätern im Homeoffice mit Kindern im Kindergarten- und Schulalter präsent. Während sich viele Eltern in der Diskussion über Infektionsschutzmaßnahmen von der Politik nicht gesehen fühlten (u. a. Andresen et al. 2020b), schufen Medien durchaus einen Raum für die Stimmen aus dem Homeoffice mit Kindern. Auch die empirischen Befragungen zu Beginn der Pandemie, so der in der KiCo-Studie entstandene Eindruck, wurden primär von Eltern, insbesondere von Müttern im Homeoffice genutzt. Bislang liegen also mehr Daten über diese Gruppe erwerbstätiger Mütter und Väter vor, als über Eltern in Kurzarbeit, mit erheblichen finanziellen Engpässen oder von Selbstständigen. Hier bestehen nach wie vor Forschungslücken.

Das Zitat aus der Überschrift ist symbolisch für die Stimmung im Homeoffice. Primär können wir über diese Gruppe und deren Krisenthemen anhand der KiCo-Studie Auskunft geben. Homeoffice mit Kindern als „Zähneputzen mit Nutella“ zu beschreiben, verweist auf das Gefühl der Vergeblichkeit des Handelns. Möglicherweise liegt darin ein übergreifender Kern pandemiespezifischen Krisenerlebens, der nicht nur diese Gruppe betrifft: Das Gefühl permanent tätig zu sein und unter Handlungsdruck zu stehen, ohne ein zufriedenstellendes Ergebnis zu erhalten.

## Zum Forschungsstand: Familien am Anfang der Pandemie

Die internationale Forschungslage ist einerseits inzwischen fast unübersichtlich und die Anzahl der allein englischsprachigen Publikationen in Zeitschriften verschiedener Disziplinen kaum mehr zu überschauen. Andererseits zeigen sich gut identifizierbare Schwerpunkte der wissenschaftlichen Betrachtung von Familien in der Pandemie, die wichtige Aspekte sichtbar machen. Gleichwohl wäre zu prüfen, welche familientheoretisch relevanten Bereiche eher marginal behandelt werden, auch weil der Zugang zu Daten erschwert ist.

Insgesamt scheint in der internationalen empirischen Forschung das Interesse an Familien, ihrer Lebenslage und ihrem Wohlbefinden in der Pandemie groß zu sein. Eine exemplarisch durchgeführte Auszählung von sozialwissenschaftlichen Artikeln im Januar 2022, die im Web of Sciences gelistet sind, vermittelt einen groben quantitativen Eindruck vom Publikationsgeschehen. Die Schlagwortverbindung „Education & COVID-19“ ergibt für das Jahr 2020 bereits 3989 Beiträge, in 2021 sind es 9537 Artikel. In der internationalen Literatur wird statt „Homeoffice“ der Begriff „Work from Home“ verwendet. Hier hat die Verbindung von „Work from Home“ & „COVID-19“ ohne disziplinäre Eingrenzungen für 2020 565 Artikel und für 2021 1419 Artikel erbracht. Nicht alle Beiträge basieren auf neuen empirischen Erhebungen, es finden sich auch Kommentare oder Review-Artikel sowie die Aufbereitung von empirischen Erkenntnissen über die Wirkungen anderer Krisen von internationaler Reichweite und Schlussfolgerungen für die COVID-19-Situation.

Unter der Verschlagwortung *Erziehung unter Bedingungen der COVID-19-Pandemie in Familien* sind verschiedene Forschungsthemen auffindbar. Sie können hier nicht in der Tiefe und vollständigen Breite dargestellt werden. Die vorgenommene Aufbereitung dient der Sensibilisierung. Eine frühe Studie aus dem DJI befasste sich mit Erziehungsfragen aus der Sicht von Eltern, aber auch von Kindern (Langmeyer et al. 2020). Sie untersuchten Veränderungen im Familienklima, das Wohlbefinden während der Kita- und Schulschließungen sowie Potenziale, die Situation gut zu bewältigen. Wichtige erziehungstheoretische Befunde sind folgende: Nach Elternauskunft hat sich ein Viertel der Kinder einsam gefühlt, wobei sich gezeigt hat, dass der Anteil in Familien mit finanziellen Engpässen deutlich höher lag, nämlich bei knapp 50 %. Hatten die Kinder Möglichkeiten, mit Geschwistern zu spielen und weiterhin Kontakt zu den Großeltern, war das Wohlbefinden deutlich besser. Für Kinder, Eltern und das Familienklima waren regelmäßige und unterstützende Kontakte zu den Pädagog:innen in Kita und Schule wichtig. Bei häufigen Konflikten in Familien hatten die Kinder und Jugendlichen – laut Elternangaben – größere Schwierigkeiten, mit der Gesamtsituation gut zurechtzukommen.

Eine größere Forschungsthematik ist in diesem Zusammenhang das elterliche Stressempfinden einerseits und Resilienzfaktoren in und für Familien andererseits. Ersteres wird als Erklärungsfaktor herangezogen, wenn es um die Konflikt- und Gewaltpotenziale in Familien geht. Stress und Belastungen in der Erziehung hängen mit ökonomischen Engpässen, verschärft im ersten Lockdown, zusammen (Langmeyer et al. 2020). Schon vor 2020 lagen auch für Deutschland empirische Befunde zu Wirkungen knapper Ressourcen bei Einkommen, Wohnraum, elterlicher Bildung auf Erziehung und Bildung im Familienalltag vor (u. a. Autorengruppe Bildungsberichterstattung 2020). Kinder und Jugendliche aus einkommensarmen Familien hatten seit dem ersten Lockdown erhebliche Nachteile, weil u. a. der Zugang zur technischen Infrastruktur fehlte. Auch enge Wohnverhältnisse haben das Stresserleben erhöht mit Auswirkungen auf die Erziehungspraktiken und die Beziehungsqualität zwischen Eltern und Kindern (Eckardt 2021).

Eine Studie von Lawson et al. (2020) aus den USA zeigt, dass der Jobverlust eines Elternteils in der Pandemie ein signifikanter Risikofaktor für eine erhöhte Prävalenz von Kindeswohlgefährdung darstellt und insgesamt eine Beeinträchtigung des Familien- und Erziehungsklimas vorkommen kann (Hupkau und Petrongolo 2020). Internationale Studien zeigen zudem eine Zunahme von Notrufen aufgrund häuslicher Gewalt im ersten Monat des Lockdowns (z. B. McCrary und Sanga 2021). Cappa und Jijon (2021) treffen auf Basis eines Literaturreviews folgende Aussagen: In vielen Ländern gab es einen Rückgang an Anzeigen bei der Polizei oder Kinderschutzbehörden, aber in einigen Ländern dafür eine Zunahme an Anrufen bei Hotlines, so auch in Deutschland (Petrowski et al. 2020). Insgesamt zeigen verschiedene Studien mit unterschiedlichen Zugängen einen signifikanten Anstieg von Gewalt in Familien, besonders während der Lockdowns (u. a. Cappa und Jijon 2021; Anderberg et al. 2021).

May et al. (2021) haben nachgewiesen, dass im weiteren Verlauf der Pandemie in Deutschland und der neuerlichen langen Schulschließung ab Dezember 2020 das elterliche Stressempfinden noch einmal deutlich gestiegen ist und dadurch Gefühle der Macht- und Hilflosigkeit vorhanden gewesen seien. Eltern mit einem höheren Bildungsniveau und passenden Arbeitszeiten zeigten jedoch ein niedrigeres Stresserleben.

Gayatri und Irawaty (2021) haben in einem Literaturreview Befunde zu Resilienz in Familien aufgelistet: eine gute und „gesunde“ Kommunikation zwischen Eltern und Kindern, gemeinsame Aktivitäten sowie familiärer Zusammenhalt und Vertrauen, das bereits vor der Pandemie hoch war.

Der Blick auf innerfamiliär stabilisierende Faktoren bedarf einer weiterführenden Analyse von unterschiedlichen Familienformen und daraus resultierenden Bedarfen an Unterstützung und stabiler Infrastruktur. Eine qualitative Studie zu alleinerziehenden Müttern mit niedrigem Einkommen aus den USA sensibilisiert dafür exemplarisch. Die interviewten Mütter berichteten, über wenig Handlungsoptionen zu verfügen, wenn es um Entscheidungen über die Betreuung ihrer Kinder während der Pandemie ging, weil ökonomische Sachzwänge den Alltag bestimmten (Radey et al. 2021).

Auch in Deutschland hat sich die Forschung mit der Situation von *Eltern im Homeoffice* und *geschlossenen Kitas und Schulen* befasst. Wir definieren diese Situation für den ersten Lockdown als Verschärfung der sogenannten „Vereinbarkeit“ und damit als spezifisches Krisenphänomen. Mit der langen Dauer der Pandemie und den inzwischen vorliegenden Erkenntnissen über Auswirkungen auf die psychische Gesundheit von Kindern und Jugendlichen sowie auf deren Bildungschancen hat sich auch die Situation in Familien und der Blick von Eltern verändert (exemplarisch dafür Ravens-Sieberer et al. 2021).

Die Herausforderungen für Eltern, Erwerbsarbeit und Karriere mit dem Familienleben und Kindern zu vereinbaren und dabei den Anforderungen im Beruf ebenso gerecht werden zu wollen, wie den Erwartungen an „gute Mutter- und Vaterschaft“ sowie an eine erfüllende Partnerschaft, sind lange vor Ausbruch der Pandemie ein empirisches Forschungsthema gewesen. Ein Fokus lag hierbei auf Erkenntnissen zu Hürden und Gelingensbedingungen für Vereinbarkeit. Zu Letzteren zählen ein verlässliches Betreuungssystem für noch nicht schulpflichtige Kinder von möglichst hoher Qualität sowie ein Angebot stabiler Betreuung und Bildung über den Mittag hinaus für Grundschulkinder und jüngere Kinder der Sekundarstufe. Doch auch eine verständnisvolle Haltung des Arbeitgebers und flexible Regelungen der Arbeitszeiten sowie möglichst kurze Wege tragen zum Gelingen von Vereinbarkeit bei (Langmeyer et al. 2020).

Anhand der Auswertungen der Panel-Studie SOEP konnte gezeigt werden, dass insbesondere Mütter und Väter von Kitakindern besonders belastet waren, weil Kinder dieser Altersgruppe kontinuierlich beaufsichtigt und beschäftigt werden müssen. Jedoch ist gerade für jüngere Kinder das elterliche Wohlbefinden sehr relevant (Huebener et al. 2020). Die Daten zeigen außerdem, dass Eltern unterschiedlich gut mit der ihnen unfreiwillig zugeschriebenen Rolle der Ersatzlehrerin, des Ersatzlehrers und den Herausforderungen der Pandemie zurechtgekommen sind (Zinn und Bayer 2021).

In Familien leben nicht nur jüngere Kinder, sondern auch Jugendliche. Die JuCo-I-Studie auf der Basis einer Befragung aus dem ersten Lockdown adressiert vorrangig das Wohlbefinden von Jugendlichen und jungen Erwachsenen. Die ersten Auswertungen verdeutlichen, dass das Wohlbefinden von Jugendlichen und jungen Erwachsenen in beengten Wohnverhältnissen erheblich eingeschränkt ist und hier auch die Reduktion des gesamten Alltags auf die häusliche Umgebung vielfach als Belastung empfunden wird (Andresen et al. 2020a, b, c, 2021; Wilmes et al. 2020; Lips 2021). Auch die SINUS-Studie zu Corona belegt, dass junge Menschen vor allem negative Assoziationen haben und von problematischen Erfahrungen mit den Einschränkungen und Gesundheitsgefahren berichten (Calmbach et al. 2020).

In der ersten Publikation von Ergebnissen der KiCo-Studie, um die es im Folgenden geht, wurden zwei Muster der Bewältigung des Lockdowns in Familien identifiziert (Andresen et al. 2020b). Ein Muster ist durch ein starkes Belastungserleben geprägt und durch Gefühle der Erschöpfung und Überforderung. Hier gibt es Hinweise darauf, dass bereits vor der Pandemie das Familienleben als anstrengend erlebt wurde, weil Erwerbsarbeit und Fürsorge schwer zu vereinbaren waren, lange Wege zurückgelegt werden mussten und zu wenig materielle Ressourcen zur Verfügung standen. Das zweite Muster gibt Hinweise auf den Wunsch zu Beginn der Pandemie, man könne auch nach der Krise einen anderen Lebensstil mit mehr Zeit, Ruhe und Ausgeglichenheit pflegen. Ein weiteres Ergebnis bezog sich auf den Eindruck der befragten Eltern, dass die Politik bei ihren Entscheidungen zum Infektionsschutz die Belange von Familien kaum berücksichtigen würde (Andresen et al. 2020b).

## Die KiCo-Studie – Eine Onlinebefragung im ersten Lockdown

### Zentrale Hypothese, Erkenntnisinteresse und Forschungsfragen

Der skizzierte Forschungsstand verdeutlicht, dass die Pandemie zügig zum Thema sozialwissenschaftlicher Forschung wurde, um dem Erleben und den Wirkungen der Infektionsschutzmaßnahmen auf die Spur zu kommen. Auch der Forschungsverbund der Universitäten Frankfurt und Hildesheim „Kindheit – Jugend – Familie in der Corona Zeit“ hat mit diesem übergeordneten Erkenntnisinteresse im Frühjahr 2020 zwei Onlinebefragungen vorbereitet und umgesetzt.^4^ Neben der oben bereits erwähnten Befragung von Jugendlichen und jungen Erwachsenen (JuCo I und II), wurde auch die Befragung von Eltern mit Kindern unter 15 Jahren (KiCo) realisiert.^5^

Für die Studie KiCo Studie wurde eine Haupthypothese aufgestellt: Der Alltag in Familien hat sich für Eltern(teile) und jedes einzelne Kind mit Beginn der Infektionsschutzmaßnahmen im Vergleich zu der Zeit vor der Pandemie erheblich verändert. Mögliche Veränderungen wurden anhand der Selbstauskunft zu Einzelitems vor und nach der Pandemie gemessen.

Ausgehend von der Veränderungshypothese lagen der Studie folgende einzelne Erkenntnisinteressen zugrunde:

- Erkenntnisse über Veränderungen im Familienalltag zum Erhebungszeitpunkt (erster Lockdown),
- über die Situation der Kinder in der Familie,
- über Herausforderungen in der Erziehung und bezogen auf Bildung,
- darüber, welche Sorgen sich Eltern(teile) machen,
- über Unterschiede zwischen dem Erleben der Kinder und der Erwachsenen in Familien,
- über Unterschiede zwischen der Zeit vor Ausbruch der Pandemie und des ersten Lockdowns und Identifikation von Ungleichheitsphänomenen,
- Identifikation unterschiedlicher Formen der Bewältigung der COVID-19-Auswirkungen auf Familie.

Daran schließen folgende Forschungsfragen an:

- Wie schätzen Eltern ihre eigene Situation und die ihrer Kinder während des Lockdowns ein?
- Welche besonderen Belastungen erleben Eltern während der ersten Wochen der Pandemie?
- Welche Sorgen haben Eltern bezogen auf sich und auf jedes einzelne ihrer Kinder?
- Inwieweit hat sich der Alltag für die Eltern und für jedes einzelne ihrer Kinder verändert?

Die Teilnehmer:innen der Studie wurden gebeten, über sich selbst, die Situation in der Familie sowie über jedes einzelne Kind Auskunft zu geben.

### Erhebungsinstrument und Feldzugang

#### Fragebogen mit Kommentarfeld

Die Studie wurde quantitativ angelegt. Es wurde ein Fragebogen erstellt, der sowohl international erprobte Fragen und Skalen zur Messung des Wohlbefindens beinhaltet als auch Fragen zum Erleben der aktuellen Situation (Andresen und Möller 2019; Andresen et al. 2020b). Am Ende des Fragebogens befand sich ein Kommentarfeld, das wie folgt eingeleitet wurde:

Vielen Dank für die Mühe, die Sie sich beim Ausfüllen dieses Fragebogens gemacht haben! Wenn Ihnen noch was eingefallen ist, etwas im Fragebogen nicht angesprochen war, freuen wir uns über Ihre Anmerkungen.

Diese Möglichkeit nutzten mit über 5000 Kommentaren sehr viele Personen.

#### Feldzugang

Der Link zur Online-Umfrage wurde nach dem Schneeballprinzip verteilt. Einzelne Personen und Netzwerke wurden aktiv ausgewählt, um den Aufruf zur Teilnahme zu verbreiten, wiederum mit der Bitte, diesen weiterzuleiten. Zusätzlich veröffentlichten beide beteiligten Universitäten eine Pressemitteilung, um die Umfrage in den Massenmedien zu verbreiten. Es ist daher nicht mehr nachvollziehbar, welche Wege die Verbreitung des Links genommen hat. Repräsentativität kann bei einer so offenen Einladung nicht erreicht werden. Das Ziel war vielmehr, möglichst viele Menschen mit der Umfrage anzusprechen. Innerhalb von 10 Tagen (vom 24.04.2020 bis 03.05.2020) beteiligten sich mehr als 25.000 Menschen aus allen Bundesländern an der Studie und beantworteten mindestens 95 % des Fragebogens^6^.

### Sample

Durch die Onlinebefragung wurden 25.177 Personen aus allen deutschen Bundesländern, die den Fragebogen für sich und ihre Kinder nahezu vollständig ausgefüllt haben, erreicht. In dieser Auswertung beschränken wir uns auf die Kommentare von 5075 Personen.

Hauptsächlich haben Mütter (rund 93 %) zwischen 30 und 50 Jahren Kommentare hinterlassen, die mehrheitlich berufstätig waren (87,7 %). 54,6 % befanden sich im Homeoffice, 26 % gingen ab und zu zur Arbeitsstelle. 18 % waren zum Zeitpunkt der Befragung durch Elternzeit oder Sonderurlaub freigestellt, 9,4 % befanden sich in Kurzarbeit (Tab. 1). Auf einen Migrationshintergrund der Befragten lässt sich nicht direkt schließen, einen Hinweis bieten die Sprachen, die zu Hause gesprochen werden. Mindestens eine weitere Sprache zu Hause zu sprechen gaben 12,3 % an.

**Table 1:** 

	Häufigkeit	Prozent
Homeoffice	2438	47,9
Gelegentliches Aufsuchen der Arbeitsstelle	1161	26,0
Arbeitssituation unverändert	884	19,8
Freistellung (inkl. Sonderurlaub, Elternzeit)	806	18,0
Kurzarbeit	419	9,4
Corona-Unterstützungsleistungen	42	0,9

Die meisten kommentierenden Personen gaben an, verheiratet zu sein (78,9 %) oder die Kinder mit jemandem gemeinsam zu erziehen (11,3 %). 7,9 % sind alleinerziehend.

In knapp der Hälfte der Haushalte (49,6 %) lebten 4 Personen. In 3‑Personen-Haushalten lebten 29,3 %, in 5‑Personen-Haushalten 13,4 %. In den meisten Fällen (53,0 %) lebten zwei Kinder unter 15 Jahren in den Haushalten, in rund 33,2 % lebte ein Kind. 13,8 % der Personen, die Kommentare hinterlassen hatten, lebten mit drei oder mehr Kindern unter 15 Jahren zusammen (s. Tab. 2).

**Table 2:** 

	Häufigkeit	Prozent
1 Kind	1692	33,2
2 Kinder	2699	53,0
3 Kinder	591	11,6
4 Kinder	95	1,9
5 Kinder	17	0,3

Unabhängig davon, wie viele Kinder in den jeweiligen Haushalten lebten, ergab sich, dass das jüngste Kind durchschnittlich etwa 5 Jahre alt war, im Falle eines zweiten Kindes war dieses knapp 8 Jahre, das dritte Kind 9,5 Jahre, das vierte Kind 11 Jahre und das fünfte Kind 13 Jahre alt. Damit sind die Kinder der kommentierenden Personen etwa ein Jahr älter als im Gesamtsample. Insgesamt lassen sich keine signifikanten Unterschiede zwischen den Merkmalen des Gesamtsamples und der kommentierenden Personen feststellen.

Diese Informationen sollten im Rahmen dieses Beitrags einen Überblick über das erreichte Sample geben, sodass ihre Reichweite, aber insbesondere auch ihre Grenzen deutlich werden. Zusammenfassend handelt es sich um ein nicht repräsentatives Sample mit spezifischen Schwerpunkten. Im dem hier relevanten Sample handelt es sich vor allem um erwerbstätige Mütter, von denen etwa die Hälfte im Homeoffice arbeitet. Diese Ausrichtung ist auch bei der Auswertung der rund 5000 Kommentare kritisch zu reflektieren.

### Auswertung der Freitexte

Die Auswertung nach der strukturierenden Inhaltsanalyse (Kuckartz 2016) erfolgte nach einem an den einzelnen Forschungsfragen orientierten Kategoriensystem. Für diesen Beitrag wurde mit der deduktiven Kategorie „Krise“ gearbeitet sowie mit drei Unterkategorien, nämlich „Belastung(en)“, „Chance“ und „Glück“.

Ein erster Überblick über das qualitative Material vermittelt sich über die Anzahl der Nennungen: So finden sich 134 Nennungen des Begriffs Krise als vorab gewählte Hauptkategorie, 438 Nennungen des Begriffs Belastung(en), 200 Nennungen des Begriffs Glück (glücklich) sowie 33 Nennungen des Begriffs Chance. Vertieft ausgewertet wurden die knapp 110 Einzelkommentare, in denen der Krisenbegriff auftauchte.^7^ Fünf markante Bezugnahmen auf die Krise konnten rekonstruiert werden: die Dauer der Krise (Zeit), Krise als Chance für gesellschaftlichen Wandel, Krise als Chance für das Glück in der Familie, die Leidtragenden der Krise, die Krise der demokratischen Gesellschaft.

## Krisenthemen in Familien zu Beginn der COVID-19-Pandemie

Im öffentlichen und medial vermittelten Diskurs tauchte der Begriff der Krise seit der ersten Wahrnehmung, dass COVID-19 auch Auswirkungen jenseits von Wuhan in China haben würde, und der ersten Infektionsschutzmaßnahmen auf. Wir haben keine Festlegung des Begriffs Krise vorgenommen, um uns für die Deutungen in den Kommentaren offen zu halten. Das Narrativ der Corona-Krise hat sich bis in die Gegenwart gehalten und findet auf nahezu alle gesellschaftlichen Bereiche Anwendung (Kühne et al. 2020).

Nach der vertieften Analyse und der Rekonstruktion der fünf induktiven Kategorien – Dauer der Krise (Zeit), Krise als Chance für gesellschaftlichen Wandel, Krise als Chance für das Glück in der Familie, die Leidtragenden der Krise, die Krise der demokratischen Gesellschaft – wurde eine gezielte Wortsuche vorgenommen. Dies resultiert aus dem ersten, aus unserer Sicht unerwarteten Ergebnis: Krise wurde in den ersten Wochen nicht mit dem Infektionsgeschehen, mit Erkrankungen und Tod sowie der Überlastung des Gesundheitssystems in Verbindung gebracht. In den 5000 Kommentaren aus den ersten Wochen der Pandemie und im Anschluss an Berichte über die hohe Todesrate etwa in Italien und an die aufrüttelnden Bilder der Särge in Bergamo tauchte der Begriff *Erkrankung* mit 45 Nennungen, *Infektionen* mit 4 Nennungen, *Tod* bzw. *Todesfälle* mit 10 Nennungen, *Krankenhaus* mit 34 Nennungen und *Überlastung des Gesundheitssystems* mit 6 Nennungen auf. Über das Krankenhaus wurde vornehmlich als Arbeitsplatz der teilnehmenden Person berichtet. Eine mit Gesundheit und COVID-19 verbundene Sorge wurde in den Kommentaren allerdings mit der Situation der Großeltern in Verbindung gebracht.^8^ Insgesamt aber war zu diesem Zeitpunkt die Krise als Thema in Familien noch nicht mit Erkrankung, Sterblichkeit und dem Gesundheitssystem verbunden, vielmehr wurde sie auf die eigene Situation bezogen.

Im Folgenden sollen die fünf Bezugnahmen anhand ausgewählter Zitate aus dem empirischen Material vorgestellt werden.

### Dauer der Krise

Belastung durch homeoffice, plus kind, plus kochen, plus putzen ist zu viel. Es wird immer schwieriger, tagesabläufe und strukturen einzuhalten, motivation, sich um schulaufgaben zu kümmern, sinkt seitens des kindes immer mehr, das ging in den ersten wochen besser. Jetzt stellt sich müdigkeit und unzufriedenheit ein. Schule plus freunde werden stark vermisst, laune sinkt, eltern können das nicht ersetzen. Arbeit bleibt stehn, druck steigt, alles wird immer schwieriger, unter einen hut zu bringen, je länger krise dauert. Kräfte verringern sich. Sand im getriebe. Immer mehr bleibt auf strecke, hausarbeit, sport, arbeit … und immer bleibt was auf der strecke, nie das gefühl, fertig zu sein oder etwas geschafft zu haben. Die berge des unerledigten oder mangelhaft erledigten türmen sich auf.^9^

Dieser Kommentar steht für die Wahrnehmung einer mit der Zeit wachsenden Belastung von Müttern, Vätern und ihren Kindern. Je länger die Krise dauere, je schwieriger sei es, den Alltag und die Strukturierung des Tagesablaufs zu bewältigen und die Motivation aufrecht zu halten. Diese Einschätzung wird für alle Familienmitglieder getroffen. Hier kommt auch eine Vielzahl an körperlichen und emotionalen Belastungen zur Sprache, über die weitere Krisenphänomene angekündigt werden. In einem anderen Kommentar heißt es dazu: „Die Psychologen werden nach dieser ganzen Krise Hochkonjunktur haben.“ Hier zeigt sich auch, dass zum Zeitpunkt der Erhebung im Frühjahr 2020 noch davon ausgegangen wurde, dass es eine kurzfristige Belastung und Ausnahmesituation sei, in der Viele an ihre Grenzen und womöglich darüber hinaus gekommen sind. Mit dem Ende dieser Zeit müsse das Erlebte aufgearbeitet werden, um schließlich wieder in einen „normalen“ Familienalltag zurückzukehren.

Doch die bis dahin verstrichene Zeit – etwa 6 bis 8 Wochen – wurde auch bereits mit positiven Gewöhnungseffekten beschrieben. Das folgende Zitat gibt wieder, dass für manche Eltern gerade die ersten Wochen als extrem krisenhaft erlebt wurden, dann aber Entspannung eintrat:

Die ersten 4 Wochen der Krise ging es mir sehr schlecht, ich musste mich zwischen Familie und Beruf entscheiden. Hätte ich diesen Fragebogen früher ausgefüllt, wäre er anders ausgefallen.

Das Zitat weist bereits auf Ressourcen hin, wenn die Möglichkeit besteht, sich zwischen zwei Optionen der Prioritätensetzung zu entscheiden.

Mit der Dauer der Krise gehen auch Zukunftsängste einher. Diese beziehen sich auf die berufliche Zukunft und das damit verbundene Einkommen sowie auf die zukünftige Bildungsbiografie der Kinder. Für Ersteres steht das folgende Zitat, über das auch die ökonomische Abhängigkeit der von der Politik unterschiedlich umgesetzten Unterstützungsmaßnahmen deutlich:

Da in Hessen selbstständige Einzelunternehmer nur mit ihren Betriebskosten unterstützt werden, mache ich mir existenzielle Sorgen. Ich habe mehr Zeit für die Kinder, das finde ich schön, aber wie es beruflich weitergehen wird, wenn die Krise länger andauert, macht mir große Sorgen. Deshalb habe ich die Selbstständigkeit bei der Berufstätigkeit extra herausgehoben; auch um darauf hinzuweisen, dass Selbstständige Angestellten in dieser Situation nicht gleichgestellt sind.

Darüber hinaus wird in dem Zitat deutlich, dass nicht nur Familien, die bereits vor der Pandemie von Armut betroffen oder armutsgefährdet waren, existenzielle Sorgen und Ängste mit sich tragen, sondern deutlich mehr Bevölkerungsgruppen betroffen sind. Jedoch, und auch das zeichnet sich hier ab, fühlte sich niemand ausreichend wahrgenommen, sodass der Eindruck entstehen konnte, man selbst sei am dramatischsten betroffen.

Der Blick auf die zu Beginn der COVID-19-Pandemie bereits als langwierig angesehene Krise sensibilisiert für die unterschiedlichen Einkommens- und Belastungssituationen in Familien und das obige Zitat verweist zugleich auf das zum Teil positive Erleben des Familienalltags, weil es mehr gemeinsame Zeit gibt (Andresen et al. 2020b). Die Sorge um Berufszweige wird auch mit dem Ende der Krise in Verbindung gebracht, wie das letzte Zitat in diesem Abschnitt von einer Musikerin verdeutlicht:

Ich arbeite als freiberufliche Sängerin. Das komplette Berufsverbot und die Sorge, dass mein Berufsfeld nach dieser Krise am Boden liegt, sind groß.

Die hier dargestellten Befunde erwecken möglicherweise zunächst den Eindruck, das Krisenerleben konzentriere sich auf wirtschaftliche und berufliche Themen der Eltern. Doch dass dies weit darüber hinausgeht, zeigen die weiteren Kategorien.

### Krise als Chance für gesellschaftlichen Wandel

Die Thematisierung von der Krise als Chance tauchte gerade in den ersten Monaten vielfach in den öffentlichen Diskussionen über COVID-19 auf. Dabei geht es um allgemeine gesellschaftliche Veränderungen, um die Haltung zum Konsum, um politische Prozesse. Eltern aber artikulieren insbesondere die Hoffnung, dass die Krise zur Verbesserung des Bildungssystems genutzt werden könnte. Den Ausgangspunkt für diese Thematisierungsweise bildet allerdings die Kritik an Defiziten sowie am Umgang der Schulen mit den Maßnahmen. Hier sind vor allem die versäumte Digitalisierung, geringe Investitionen und der Föderalismus genannt worden.

Eine andere Person bringt ihre Hoffnung auf Veränderung zugunsten der Kinder und Jugendlichen deutlich zum Ausdruck und formuliert damit einhergehend einen klaren Appell an politische Entscheidungsträger:innen:

Und ich hoffe, dass durch diese Krise eine Chance genutzt wird und sich einiges am Bildungssystem verändert! Potenziale zu entfalten, statt allen dasselbe einzutrichtern!

### Krise der Demokratie

Anschließend an bildungspolitische Forderungen und Wünsche, befassen sich viele Kommentare mit politisch gedeuteten Krisenphänomenen. Neben anerkennenden Äußerungen zu einem insgesamt guten Krisenmanagement, wird der Politik Perspektivlosigkeit vorgeworfen und daran der Kern der Krise festgemacht.

Die Perspektivlosigkeit, in der uns die Politik lässt, ist schwer. Was antwortet man den Kindern, wann sie mal wieder andere Kinder treffen können? Ganz zu schweigen von Kindergarten oder Schule. Zudem ist mir unverständlich, wie Friseure und Geschäfte öffnen sollen, wenn es keine Kinderbetreuung gibt.

Damit einher geht zugleich eine Kritik an der Kommunikation, weil „die Politik“ für Eltern keine Perspektive, keinen Endpunkt der Krise festmachen kann und sich dadurch der Eindruck verfestigt, Politik habe Familien vergessen und sei nicht für deren Situation und Alltag sensibilisiert. An dieser Stelle wird auch deutlich, dass Politik bzw. politische Entscheidungen einen unmittelbaren und direkten Einfluss auf das Leben aller Familien hat, wie es zuvor in seiner Wirkungsmacht kaum vorkam. Politik wurde so auch Gegenstand von Erziehung. In ihrer Rolle als Eltern müssen sie ihren Kindern mitunter Dinge erklären, die sie selbst zum Teil nicht durchblicken, deren Sinn sie nicht verstehen und absehen können. Die Unsicherheiten der Eltern können sich auch auf die Kinder übertragen. Für einige Eltern ist die Vermittlung eigener Unsicherheiten oder der Umgang damit gegenüber ihren Kindern sicherlich eine neue Situation. Es entstehen Situationen, Umstände, Regeln und Verbote, die Eltern womöglich schwer rechtfertigen können. Dass dies eine große Herausforderung in der Erziehung sein kann, wird in dem oberen Zitat benannt.

Die Themenfelder Verunsicherung, Manipulation und die Fragen nach dem sozialen Frieden in der Gesellschaft treiben manche Studienteilnehmer:innen auch in ihrer Elternrolle um:

Ich sehe in meinem Umfeld verstärkt Tendenzen, dass Menschen sich nicht gut informiert und manipuliert fühlen. Der sozialen Frieden ist in meiner Sicht stark gefährdet. Die Lösung dieser Krise braucht eine vielschichtige und demokratische Diskussion, die es im Moment an vielen Stellen nicht gibt. Das schürt Ängste und hilft nicht bei der Lösung der anstehenden Probleme.

Dass in diesem Fall zwei Gruppierungen aufgemacht werden – ich und die anderen Menschen, die nicht gut informiert sind und sich von außen gesteuert fühlen – weist bereits im Frühjahr auf die Wahrnehmung einer möglichen Spaltung der Gesellschaft hin, wie es besonders im Jahr 2021 hinsichtlich verschieden motivierter Proteste im öffentlichen Diskurs thematisiert wurde. In diesem Zitat lässt sich auch eine Kritik an der politischen Kommunikation und dem demokratischen Prozess ablesen. Ängste entstünden, wenn Menschen in ihren Lebenssituationen nicht abgeholt würden und bloß vor getroffene Entscheidungen gestellt würden, ohne einen demokratischen Dialog.

### Krise als Chance für die Familie

Bereits eine erste Auswertung der KiCo-Studie hatte ergeben, dass es auch Familien im ersten Lockdown gab, die sehr gut mit der neuen Situation zurechtkamen und diese vielfach als glückliche Zeit erlebt haben (Andresen et al. 2020b). Hier spielten weggefallene Anfahrtswege zur Arbeit, Zeitgewinn durch ausgefallene Freizeitaktivitäten, mehr Ruhe im Alltag und gemeinsame neu entdeckte Interessen und Aktivitäten eine Rolle. Häufig war das positive Erleben an räumliche, finanzielle und soziale Ressourcen gekoppelt. Dieses Phänomen lässt sich auch in Zusammenhang mit der Thematisierung von Krise nachweisen:

Die Krise bietet uns eine intensive Zeit als Familie zu erleben – unbezahlbare Augenblicke, Momente und Erlebnisse. Zeit für Sachen, die wir sonst nicht hatten und gemeinsam neu entdecken.

Diese ersten Wochen der Pandemie haben einigen Familien auch ökonomische Spielräume gebracht, einzelne Familienmitglieder von Zwängen befreit und Handlungsspielräume eröffnet. Dies thematisiert auch diese Person als Chance im Hier und Heute ebenso wie für die Zukunft:

Durch die Corona-Krise spart man Sprit, unnötige Fahrzeiten (2 h) auf der Straße, schont die Umwelt, braucht weniger Schmincke/Kosmetik, hat mehr Zeit, in die Natur zu gehen, mehr Zeit mit Kind, endlich raus aus dem Hamsterrad und Spagat zwischen Kita, Job, Kind, Haushalt, gesellschaftlichen Verpflichtungen/Druck, Traditionsunternehmen sind gezwungen, endlich Homeoffice zu ermöglichen und die Digitalisierung voranzutreiben.

In vielen Familien sind Belastungen aus der Zeit vor der Pandemie aber keineswegs weniger geworden, sondern sie haben sich teilweise verstärkt, beispielsweise die Belastungen durch Armut. Vor diesem Hintergrund ist der abschließende Kommentar einer Alleinerziehenden aufschlussreich, weil sie darauf aufmerksam macht, dass viele Hürden der Alltagsbewältigung in der COVID-19-Pandemie für Alleinerziehende auch bereits vorher vorhanden waren:

Als Alleinerziehende ist man daran gewöhnt, seinen Alltag allein zu bewältigen. Somit stemmen wir die Krise vermutlich am besten.

Neben ihrer Vulnerabilität im gesellschaftlichen Gefüge, stellt die Mutter hier ihre Kompetenzen hervor, die sie im Umgang mit dieser Vulnerabilität, an die sie „gewöhnt“ ist, bereits erworben hat. Nach ihrer Auffassung sind Alleinerziehende demnach krisenerprobt und zu einem gewissen Grad krisenfest. Dieser Blick könnte eine neue Dimension in die Diskussion um besonders vulnerable Eltern eröffnen. Denn es ließe sich demnach argumentieren, die Pandemie habe zuvor wenig vulnerable Eltern in eine Lage gebracht, in denen sich z. B. Alleinerziehende „schon immer“ befinden.

### Leidtragende der Krise

Viele Kommentare befassen sich mit den Leidtragenden der Krise. Hier werden vor allem die eigenen Kinder bzw. Kinder insgesamt genannt. Dabei wird der Politik in Deutschland Kinderfeindlichkeit attestiert und die Wahrnehmung als Krisenphänomen thematisiert, die Politik orientiere sich nicht an den Bedürfnissen von Kindern und Jugendlichen.

Es ist so traurig, dass unsere Kinder keine oder kaum Lobby haben – aus diesem Grund sind die Kinder die großen Verlierer der Krise. Mit großer Sorge schauen wir auf die Zukunft und fragen uns jetzt noch mehr als vorher, warum kein Geld für Schulen, Lehrmittel etc. da ist, aber Milliarden, um die Wirtschaft zu retten (was natürlich sein muss nach dem Lockdown). Fazit Geld ist genug da – aber leider wird es nicht so gerne für die Kinder verwendet … traurig.

Ein Kommentar adressiert die Qualität der Notbetreuung und beschreibt das eigene Zögern, die Kinder dort unterzubringen, aber es perspektivisch nicht ohne diese zu schaffen:

Die Doppelbelastung mit Kinderbetreuung und Vollzeitarbeit ist enorm. Trotzdem bleibt das schlechte Gewissen, die Kinder in der Krise in die Notbetreuung zu schicken. Wir haben uns bis jetzt nicht getraut, aber wahrscheinlich müssen die Kinder ab Mai dann zurück in die Kita. Anders ist es einfach nicht zu schaffen.

Die Sorge um die Kinder wird an dieser Stelle mit der schwierigen Vereinbarkeit von Sorge- und Erwerbsarbeit bzw. der Doppelbelastung in Verbindung gebracht.

Neben den Kindern werden vor allem die Mütter als Leidtragende der Krise bezeichnet, was möglicherweise auf deren hohe Beteiligung an der Befragung zurückzuführen ist. Vor allem jedoch bringt dies ihre oftmals verzweifelte Lage zum Ausdruck.

Am Ende sind die Mütter wieder diejenigen, auf deren Schultern diese Krise ausgetragen wird.

Das abschließende Zitat verweist auf eine Gruppe von Kindern und ihren Familien, für die der Wegfall der Infrastruktur von Anfang an eine außerordentliche Herausforderung darstellte, weil sie aufgrund besonderer Bedürfnisse auf eine gute Unterstützung und Versorgung angewiesen sind. Kinder mit Behinderung und ihre Familien waren lange nicht im Fokus der Aufmerksamkeit und sind in den ersten Abwägungsprozessen zwischen Infektionsschutz und Öffnung selten berücksichtigt worden.

Wir sind in der Corona-Krise in eine besonders schwierige Lage gekommen. Unser ältester Sohn ist auf einen Rollstuhl angewiesen. Die Betreuung und Pflege, die ja auch von seiner Schule mit übernommen wurde, ist komplett weggefallen. Leider übernehmen derzeit auch die Großeltern einen Teil der Betreuung. Wir haben gar keine andere Wahl!

Dieses Zitat verweist sehr deutlich darauf, dass im öffentlichen Diskurs, in der Forschung und bezogen auf politische Maßnahmen verschiedene Familienkonstellationen und Bedarfe nicht im Fokus waren, dazu gehörten Familien mit beeinträchtigten Mitgliedern. Darauf haben zur Jahreswende 2020/2021 Beiträge aus dem Beirat für Familienfragen beim BMFSFJ hingewiesen.

## „… wie Zähneputzen mit Nutella“ – Diskussion der Ergebnisse und Fazit

An der hier im Zentrum stehenden Studie KiCo haben sich vor allem Mütter beteiligt, die zur Zeit der Erhebung im Homeoffice waren. Die Zusammensetzung des Samples wurde im Methoden- und Auswertungsteil beschrieben und kritisch reflektiert. An dieser Stelle sei nur darauf verwiesen, dass die Belastungen durch die Gemengelage von Anforderungen angesichts gleichzeitig bestehender Zuständigkeiten kein neues Phänomen darstellen. Für erwerbstätige Mütter und Väter geht es seit Langem um Fragen der sogenannten „Vereinbarkeit“ ihrer Verantwortung für Einkommen, Erwerbsarbeit und Karriere sowie für Betreuung und Bildung von Kindern und weiterer Familientätigkeiten.

Die Auswertung der Kommentare gibt aufschlussreiche Hinweise auf das Krisenerleben in den ersten Wochen der COVID-19-Pandemie aus der Perspektive von Eltern mit Kindern unter 15 Jahren. Persönliche Einschränkungen oder aber neue Spielräume, familiäre Perspektiven und gesellschaftliche Belange treten unter dem Begriff der Krise in eine Art Dialog.

Anfänge definieren zu wollen, birgt die Gefahr einer Idealisierung, Mythenbildung oder historischen Blindheit. Dennoch wurde hier der Blick auf eine Art Anfang gerichtet, um für die Dynamik, die Komplexität, für die Prozesshaftigkeit und Diversität des Pandemiegeschehens, von der bereits allein in Deutschland auszugehen ist, zu sensibilisieren.^10^

Die Daten der KiCo-Studie wurden im ersten Lockdown in Deutschland erhoben, sie haben damit eine begrenzte Aussagekraft darüber, wie Familien die Pandemie insgesamt erlebten. Wir haben für diesen Beitrag versucht, aus diesem „Anfangserleben“ spezifische Erkenntnismöglichkeiten zu generieren und den Blick für Veränderungen, die für Eltern, Kinder, Jugendliche und Familien in den Folgemonaten der Jahre 2020 und 2021 besonders relevant waren, zu schärfen. Die Ergebnisse verdeutlichen die Vielfalt in Familien und die Unterschiede zwischen Familien, auch in Zeiten der Pandemie. Gleichwohl lassen sich besonders hartnäckige Belastungsfaktoren, von denen nahezu alle Familien besonders betroffen sind, benennen.

Zwar wurde mit der Studie ein außerordentlich großes Sample erreicht, dennoch kann nicht von Repräsentativität gesprochen werden. Die hohe Teilnahme interpretieren wir als Bedürfnis, sich mitzuteilen und gehört zu werden. Aber es zeigt sich, dass diese Möglichkeit längst nicht alle nutzen können oder wollen.

## Footnotes

1 Ende April 2020 übernahmen in 14 % der Familien die Großeltern Betreuungsaufgaben. Bis Ende Mai stieg der Anteil kontinuierlich auf 22 % an (Langmeyer et al. 2020).

2 Im März 2020 bekamen 2,49 Mio. Arbeitnehmer:innen Kurzarbeitergeld von der Bundesagentur für Arbeit gezahlt. Der Höchststand wurde im April 2020, und damit auch zum Zeitpunkt der Befragung, erfasst: 6,83 Mio. Arbeitnehmer:innen (20,4 % aller sozialversicherungspflichtig Beschäftigten) (Bujard et al. 2020).

3 Im April 2020 mussten sich 381.000 Personen arbeitslos melden, im Mai 197.000. Insgesamt sind etwa 20 % der Arbeitslosmeldungen auf die Corona-Krise zurückzuführen (Bujard et al. 2020).

4 https://t1p.de/studien-corona.

5 Die Studien wurden von der Ethikkommission des Fachbereichs Erziehungswissenschaften der Goethe-Universität Frankfurt begutachtet und bewilligt. Vom Datenschutzbeauftragten der Stiftung Universität Hildesheim wurden die datenschutzrechtlichen Fragen geprüft.

6 Die Teilnehmer:innen konnten außerdem an einer Gutscheinverlosung teilnehmen.

7 In einigen Kommentaren tauchte Krise mehrfach auf.

8 Für Oma bzw. Großmutter gab es 128, für Opa/Großvater 82 und für Großeltern 231 Nennungen.

9 Schreibweise hier und in den folgenden Zitaten nur minimal angepasst.

10 Aufschlussreich hierfür sind die Beiträge der Mitglieder des Wissenschaftlichen Beirats für Familienfragen beim BMFSFJ (Spieß et al. 2021).
